# Decelerated lattice excitation and absence of bulk phonon modes at surfaces: Ultra-fast electron diffraction from Bi(111) surface upon fs-laser excitation

**DOI:** 10.1063/1.5128275

**Published:** 2019-11-05

**Authors:** V. Tinnemann, C. Streubühr, B. Hafke, T. Witte, A. Kalus, A. Hanisch-Blicharski, M. Ligges, P. Zhou, D. von der Linde, U. Bovensiepen, M. Horn-von Hoegen

**Affiliations:** Department of Physics and Center for Nanointegration (CENIDE), University of Duisburg-Essen, 47048 Duisburg, Germany

## Abstract

Ultrafast reflection high-energy electron diffraction is employed to follow the lattice excitation of a Bi(111) surface upon irradiation with a femtosecond laser pulse. The thermal motion of the atoms is analyzed through the Debye–Waller effect. While the Bi bulk is heated on time scales of 2 to 4 ps, we observe that the excitation of vibrational motion of the surface atoms occurs much slower with a time constant of 12 ps. This transient nonequilibrium situation is attributed to the weak coupling between bulk and surface phonon modes which hampers the energy flow between the two subsystems. From the absence of a fast component in the transient diffraction intensity, it is in addition concluded that truncated bulk phonon modes are absent at the surface.

## I. INTRODUCTION

The initial dynamics of atoms in solid states upon impulsive femtosecond laser excitation has recently attracted much attention because energy transfer processes between the electronic system and the lattice are of general importance and high technological relevance. With the advent of ultrafast transmission electron diffraction and X-ray diffraction techniques, the structural dynamics became accessible on the picosecond and femtosecond time scales through the transient changes in the diffraction patterns upon ultrashort laser excitation. Typical time scales for the energy transfer from the hot electron system to the vibrational motion of the atoms cover the range from 0.3 ps for multilayer graphene^1–4^ and Ge,^5,6^ 1 ps for Al,^7,8^ and up to 5 ps for Au.^9^

Crystalline Bi has widely been used as a prototypical system for the study of such ultrafast energy transfer processes from the initially excited electron system to the phonon system. Depending on the degree of femtosecond laser irradiation, vastly different time constants for the excitation process of the Bi lattice were observed. In the regime of strong excitation, when more than 2.5% of the valence electrons are excited, the potential energy landscape for the atomic positions is drastically changed.^10^ This results in an inverse Peierls transition and the electronic acceleration of the atomic motion which can be as fast as 180 fs.^11^ Such displacive acceleration causes strong excitation of the coherent *A*_1__*g*_-phonon mode which in addition exhibits the effect of bond softening.^12–14^ Under the conditions of weak excitation of the electron system, the potential energy landscape remains almost unchanged. The lattice is then heated on time scales of 2 to 4 ps^11,15–20^ through electron–phonon coupling in which Bi is weak compared to other materials.^21^ Thus, the coupling between the excited electron system and the lattice is well explored for the bulk of Bi.

Little, however, is known about the energy transfer processes from the bulk to the surface of a solid-state material. A crystalline surface exhibits specific electronic states and phonon modes which are confined to the surface. Often, the phonon surface states do not overlap with the projected bulk phonon states, which implies only the weak vibrational coupling of the surface atoms to the bulk. We therefore may expect that the initial structural dynamics of surface atoms is different from that of atoms in the bulk. This has been demonstrated for the monolayer adsorbate system Pb on Si(111) which exhibits very specific optical vibrational modes which were excited upon the decay of the electronic excitation.^22,23^ Via mode conversion, these optical modes subsequently decay into acoustic modes. These low frequency modes are trapped in the adsorbate layer because they do not couple to the Si substrate modes and survive for nanoseconds. Similar questions arise for the surface of a solid bulk: What is the excitation process and what are the characteristic time scales for the excitation of the thermal motion of the surface atoms? Does the surface phonon system play a major role, or do the surface atoms just follow the excitation of the bulk, with both subsystems in mutual equilibrium?

Here, we explore these fundamental questions by means of time-resolved reflection high-energy electron diffraction (RHEED) using hetero epitaxial Bi(111) films on a Si(111) substrate.^22,24–27^ The grazing incidence of the electrons ensures surface sensitivity, and only the topmost bilayer of the Bi film contributes to the RHEED pattern.^28^ The excitation of the surface lattice is followed employing the Debye–Waller effect which describes the reduction of the intensity *I*∕*I*_0_ of the diffraction spots

$$I/I_{0}=\text{exp}\;\left(-1/3\;\Delta \langle u^{2}\rangle \Delta k^{2}\right)$$ (1)

upon the change in an isotropic mean squared displacement $\Delta \langle u^{2}\rangle$ of the thermal motion of the atoms and the momentum transfer Δ*k* of the diffracted electrons. *I*_0_ is the intensity at the base temperature *T*_0_ prior to excitation with a mean squared amplitude $\left\langle u_{0}^{2}\right\rangle$. Applying the Debye model, we can correlate a change in the mean squared displacement of the surface atoms to a change in the temperature Δ*T*

$$\Delta \langle u^{2}\rangle =\frac{3\hbar^{2}\Delta T}{Mk_{B}\Theta_{D,\text{surf}}^{2}},$$ (2)

where $\Theta_{D,\text{surf}}$ is the surface Debye temperature in the framework of individual harmonic oscillators [$\Theta_{D,\text{surf}}=47\;K$ for the Bi(111) surface^29,30^] and *M* the atomic mass of Bi.

## II. EXPERIMENTAL SETUP

**FIG. 1.**
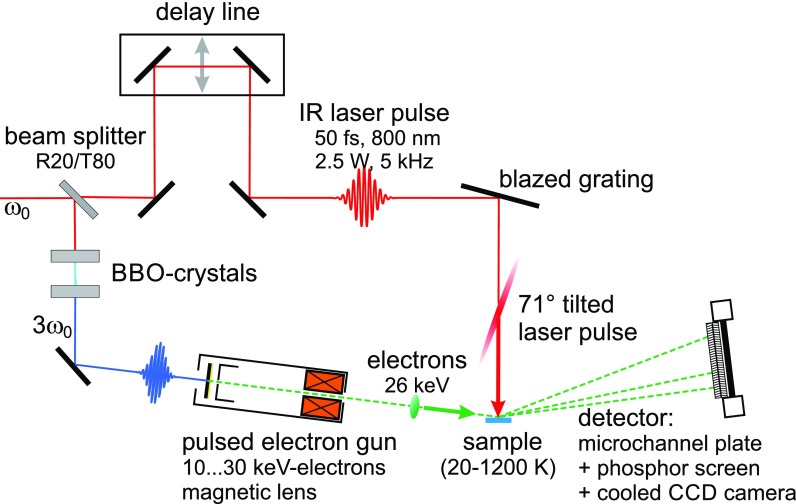
Setup of the pump-probe experiment. The sample is excited through a femtosecond-IR laser pulse and subsequently probed by an ultrashort electron pulse. The time delay *t* is varied by an optomechanical delay line. The velocity mismatch between electrons at grazing incidence and the laser pulse at normal incidence is compensated by tilting the laser pulse front by 71°. The sample is prepared *in situ* under ultrahigh vacuum conditions.

Time-resolved RHEED experiments are performed under ultrahigh vacuum conditions at a base pressure below $2\times 10^{-10}\;\text{mbar}$. The sample is an epitaxial, 8 nm thin Bi(111) film grown *in situ* on a clean Si(111)–(7 × 7) reconstructed substrate as described elsewhere in detail.^31,32^ The experimental setup is sketched in Fig. 1. A regenerative Ti:Sapphire laser system provides laser pulses of 0.5 mJ energy with a central wavelength of 800 nm and a duration of 50 fs at a repetition rate of 5 kHz. The third harmonic of the fundamental generates electron pulses via photoelectron emission from a back-illuminated gold photo cathode.^33^ The electrons are accelerated to 26 keV and focused by a magnetic lens. Diffraction occurs at the sample under a grazing incidence of 3.4°. The diffraction pattern is amplified by a microchannel plate (MCP), detected by a phosphor screen, and recorded by a cooled CCD camera. The sample is cooled by a liquid-nitrogen cryostat to a temperature of 90 K and impulsively excited by the fundamental of the laser system under normal incidence at a pump fluence of 3 mJ∕cm^2^. The time delay between the pumping laser pulse and the probing electron pulse is varied systematically via an optomechanical delay line. To compensate the different arrival times of the electrons at the sample, the pumping laser pulse front was tilted by an angle of 71°, ensuring constant delay times across the entire sample.^34^ Details are described elsewhere.^35^ The experimental temporal resolution of better than 3 ps was, however, still governed by a small velocity mismatch.

## III. RESULTS AND DISCUSSION

**FIG. 2.**
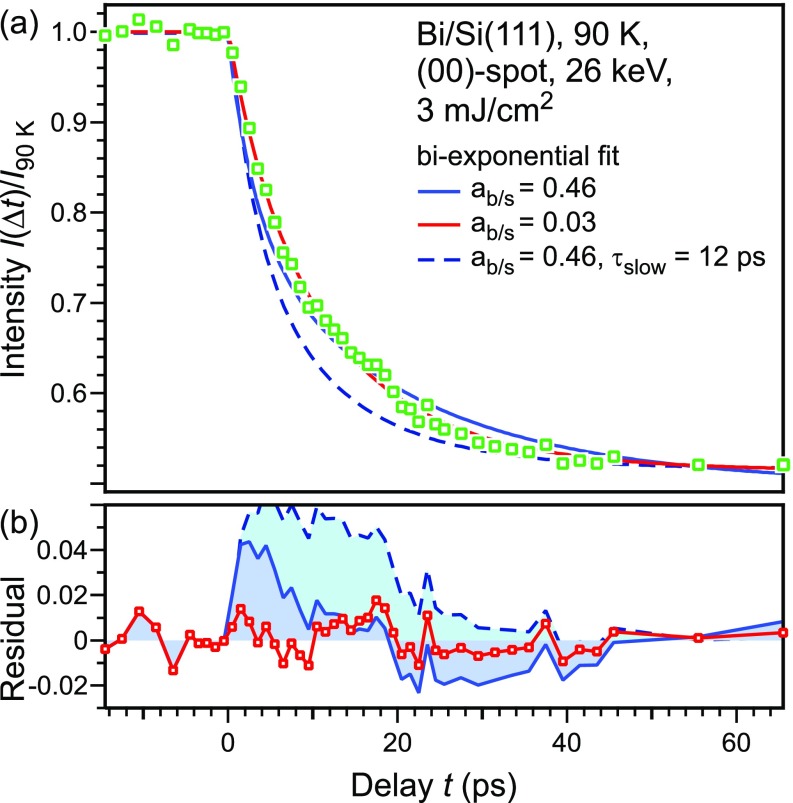
(a) Diffraction intensity of the (00)-spot is plotted as open squares as a function of the delay time *t*. The data are fitted with a biexponential decay function defined in Eq. (3) for two different ratios $a_{b/s}=\Delta \langle u_{\text{trunc},\infty }^{2}\rangle /\Delta \langle u_{\text{surf},\infty }^{2}\rangle$ of the mean squared displacements of the bulk and surface phonon modes. (b) Residuals between the fit and data.

**FIG. 3.**
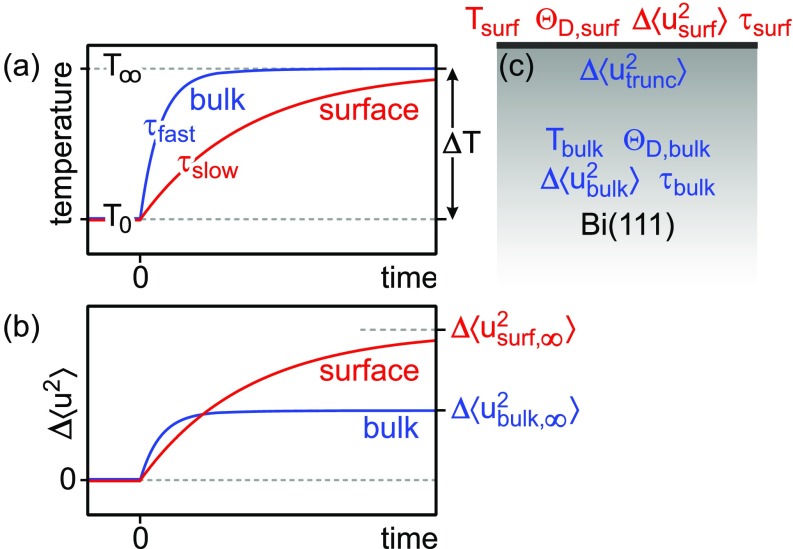
(a) Sketch of the transient rise of temperature upon impulsive excitation at *t *=* *0. Time constants are $\tau_{\text{bulk}}$ and $\tau_{\text{surf}}$ for bulk and surface temperatures, respectively. (b) Transient change in squared vibrational amplitude $\Delta \langle u^{2}\rangle$ for the bulk and surface. Due to missing bonds, the surface atoms exhibit a larger change in squared amplitude. (c) Schematics of the bulk/surface system with the corresponding parameters.

The intensity of diffraction spots is analyzed as a function of delay time *t* between the laser pump and the electron probe pulse. Figure 2(a) depicts the transient diffraction intensity of the (00)-spot (open squares) for delay times from −10 to 65 ps. For negative delays *t *<* *0, the diffraction intensity remains constant because the sample is probed prior to excitation. For positive delay times *t *>* *0, the intensity drop is caused by the Debye–Waller effect: subsequently to the laser excitation, the phonon system is excited, resulting in an increase in the vibrational amplitude of the atoms as given by Eq. (1). In a previous analysis, the initial transient intensity drop of all diffraction spots in the RHEED pattern was described by monoexponential decay functions.^19,36^ This yielded distinctly different time constants in the range of 5 to 12 ps. All of this could be explained by the inherent nonlinearity of the exponential function: for large momentum transfers, the spot intensity decreased more than 90%, which then is associated with an apparently faster excitation. The consideration of this effect—for all spots—resulted in a consistent time constant of 12 ps for the vibrational excitation of the surface atoms.^36^ Thus, the excitation of the surface phonons is slow compared to the excitation of the bulk phonon system, which occurs within 4 ps or less.^11,19^ This situation is sketched in Fig. 3 with the transient temperatures and changes in mean squared displacements $\Delta \langle u^{2}(t)\rangle$ for the bulk and surface in blue and red curves, respectively. The decelerated excitation of the surface vibrational phonon modes was explained by the weak coupling between bulk modes and surface modes: weak overlap between the dispersion relation of projected bulk modes and surface modes prevents the effective coupling between bulk and surface vibrations.^23,37,38^ Therefore, one would expect that the surface atoms respond on two different time scales upon impulsive excitation.

Let us assume that truncated bulk phonon modes are present at the surface that are excited at a time constant of $\tau_{\text{bulk}}$ = 3 ps in addition to surface phonon modes that are excited on a slower time scale within $\tau_{\text{surf}}$ = 12 ps. We then expect a biexponential decay of the RHEED spot intensity with these two time constants: $\tau_{\text{bulk}}$ for bulk excitation and $\tau_{\text{surf}}$ for surface excitation

$$I(t)=I_{0}\cdot \;\text{exp}\;\{-\frac{1}{3}\left[\Delta \langle u_{\text{trunc}}^{2}(t)\rangle +\Delta \langle u_{\text{surf}}^{2}(t)\rangle \right]\Delta k^{2}\}$$ (3)

with the first exponential function

$$\Delta \langle u_{\text{trunc}}^{2}(t)\rangle =\Delta \langle u_{\text{trunc},\infty }^{2}\rangle \Theta (t)(1-\text{exp}\;(\frac{-t}{\tau_{\text{bulk}}}))$$ (4)

describing the transient change (time constant $\tau_{\text{bulk}}$) in the mean squared displacement $\Delta \langle u_{\text{trunc}}^{2}(t)\rangle$ of bulk modes reaching up to the surface, i.e., the so-called truncated bulk modes. The second exponential function

$$\Delta \langle u_{\text{surf}}^{2}(t)\rangle =\Delta \langle u_{\text{surf},\infty }^{2}\rangle \Theta (t)(1-\text{exp}\;(\frac{-t}{\tau_{\text{surf}}}))$$ (5)

describes the change in the mean squared displacement $\Delta \langle u_{\text{surf}}^{2}(t)\rangle$ of surface modes that are excited at a time constant $\tau_{\text{surf}}$. The values $\Delta \langle u_{\text{trunc},\infty }^{2}\rangle$ and $\Delta \langle u_{\text{surf},\infty }^{2}\rangle$ give the change in mean squared displacement for long time scales, i.e., upon equilibration of the bulk and surface phonon system. Θ(*t*) is the Heaviside function. The expected transient changes in the squared vibrational amplitudes $\Delta \langle u^{2}\rangle$ for the bulk and the surface are sketched in Fig. 3(b).

Upon thermalization at time scales $t\gg \tau_{\text{surf}}$, the bulk and the surface phonon system must exhibit the same temperature change △*T*. In the framework of the Debye model, the change in the mean squared displacement is given by Eq. (2). Considering the different Debye temperatures (harmonic oscillator model) for the surface and the bulk of $\Theta_{D,\text{surf}}=47\;K$^29,30^ and $\Theta_{D,\text{bulk}}=120\;K/\sqrt{3}$,^39^ respectively, we expect a ratio $a_{b/s}$ between the bulk and surface mean squared displacement

$$a_{b/s}=\frac{\Delta \langle u_{\text{bulk},\infty }^{2}\rangle }{\Delta \langle u_{\text{surf},\infty }^{2}\rangle }=\frac{\Theta_{D,\text{surf}}^{2}}{\Theta_{D,\text{bulk}}^{2}}\approx 0.46.$$ (6)

Let us first assume that the truncated bulk modes have the same amplitude at the surface as they have in the bulk, that is, $\Delta \langle u_{\text{bulk},\infty }^{2}\rangle =\Delta \langle u_{\text{trunc},\infty }^{2}\rangle$. Under these conditions, a biexponential fit with a ratio $a_{b/s}=\Delta \langle u_{\text{trunc},\infty }^{2}\rangle /\Delta \langle u_{\text{surf},\infty }^{2}\rangle =0.46$ should describe the transient intensity drop. The fast time constant of bulk excitation $\tau_{\text{bulk}}$ was set at 4 ps, as given by an average of the literature time constants for the bulk excitation of 3 ps convoluted with our instrumental temporal response function. The best fit gave a slow time constant of $\tau_{\text{surf}}=23\;\text{ps}$ and is shown as a solid blue line in Fig. 2(a). The residual between the fit and experimental data shown in Fig. 2(b) reveals systematic deviations: the data are not described by a value of $a_{b/s}=0.46$.

The best fit is obtained for $a_{b/s}\approx 0.03$ and $\tau_{\text{surf}}=13\;\text{ps}$, plotted as a solid red curve in Fig. 2, which essentially is a monoexponential decay. Now, the residual of the fit—shown as a red curve with open squares in Fig. 2(b)—exhibits random deviations only except for a small bump at 16 ps. This bump arises from a standing wave mode of the d=8 nm thick Bi film. The period of this acoustic mode is given by $T=2d/v_{\text{sound}}$ with $v_{\text{sound}}=1074\;m/s$. From the monoexponential decay, we have to conclude that the change in the vibrational amplitude of the bulk phonon modes must be much smaller at the surface than in the bulk. With both parameters kept constant, $\tau_{\text{surf}}=12\;\text{ps}$ and $a_{b/s}=0.46$, the agreement is worst and the data cannot be described with these parameters (dashed blue line in Fig. 2).

**FIG. 4.**
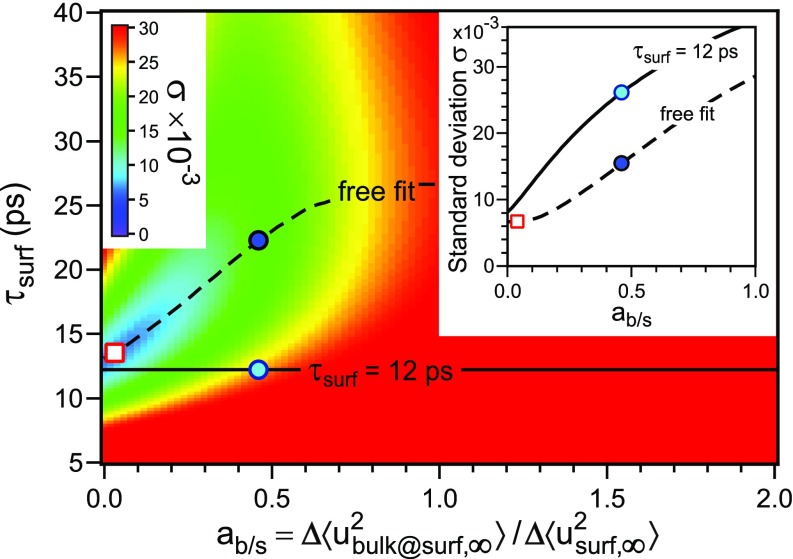
False color representation of the standard deviation of the biexponential fit as a function of $a_{b/s}$ and $\tau_{\text{surf}}$. The dashed line indicates the optimum values of $\tau_{\text{surf}}$ for each $a_{b/s}$ (free fit). In the inset, the minimum standard deviation (dashed line) and standard deviation for constant $\tau_{\text{surf}}=12\;\text{ps}$ (solid line) are plotted as a function of $a_{b/s}$.

To estimate the degree of confidence of the biexponential fit, we systematically varied the parameter *a*_b∕s_ and the time constant $\tau_{\text{surf}}$ for the slow decay of intensity. The standard deviation of this biexponential fit to the experimental data is mapped in Fig. 4 as a function of *a*_b∕s_ and $\tau_{\text{surf}}$. Blue color mapping represents the best agreement between the fit and data, and red color mapping is worst. Again, the minimum standard deviation is found for small values of *a*_b∕s_, i.e., a monoexponential behavior with no fast component.

The dashed line in Fig. 4 indicates the optimum *τ*_surf_ as a function of parameter *a*_b∕s_. With increasing *a*_b∕s_, i.e., assuming an increasing bulk contribution $\Delta \langle u_{\text{trunc},\infty }^{2}\rangle$ of the fast decay, we obtain an increasing time constant *τ*_surf_ for the slow intensity decay. This dependence confirms that the slow excitation of the vibrational motion of the surface atoms is a robust finding. At the same time, with increasing parameter *a_b_*_∕__*s*_, the standard deviation increases, too. This is shown in the inset in Fig. 4 for the minimum standard deviation (dashed line) as a function of *a*_b∕s_. We again find the best agreement between data and biexponential fit for the small values of $a_{b/s}<0.2$ as indicated by the open red square in Fig. 4. If we keep the slow time constant fixed at $\tau_{\text{surf}}$ = 12 ps, the fit becomes worse and an even larger standard deviation is obtained (solid line in the inset in Fig. 4). The two examples of Fig. 2 at *a_b_*_∕__*s*_ = 0.46 are indicated by the blue dots and exhibit a significant larger standard deviation. We therefore conclude that the increase in the mean squared displacement of bulk modes at the surface $\Delta \langle u_{\text{trunc},\infty }^{2}\rangle$ upon excitation is smaller by a factor of more than 2.5 than the corresponding increase in the mean squared displacement of the same modes $\Delta \langle u_{\text{bulk},\infty }^{2}\rangle$ in the bulk. Furthermore, the increase in the mean squared displacement $\Delta \langle u_{\text{surf},\infty }^{2}\rangle$ of the surface phonon modes is by a factor of more than 5 larger than any truncated bulk modes at the surface: surface phonons vastly dominate the thermally-activated vibrational motion of atoms at the surface and bulk modes are not observed.

## IV. CONCLUSION

Ultrafast time-resolved measurements open up a new route to determining hidden parameters—such as the coupling strength between electronic or phononic subsystems—that are not accessible for measurement under equilibrium conditions. For example, the energy transfer rate between different subsystems can be directly determined from the transient behavior of such systems following impulsive excitation. This includes electron–electron coupling,^40–43^ thermalization in the electron system,^41,44^ electron–phonon coupling,^3,45,46^ or the mode conversion within the phonon system.^2,22,23,47^ With the evolution of a nonequilibrium state in the time domain as an additional degree of experimental freedom, new insights into long-known processes can be gained.

Here, we have studied the decelerated excitation of thermal motion of surface atoms by means of time-resolved electron diffraction. We used femtosecond-laser pulses to impulsively excite a single crystalline Bi(111) film. The transient dynamics of the surface atoms was followed through the Debye–Waller effect in surface-sensitive electron diffraction. While it is well known from the literature that the bulk is heated in less than 4 ps, we observe that the excitation of vibrational motion of the surface atoms occurs much slower at a time constant of 12 ps. This behavior is attributed to the weak coupling between bulk and surface phonon modes which is explained by the weak overlap between bulk phonon bands and surface phonon modes in the phonon dispersion relation. From the absence of a fast component in the transient RHEED intensity drop, we additionally have to conclude that truncated bulk phonon modes are absent at the surface.
